# A Dispersion Corrected DFT Investigation of the Inclusion Complexation of Dexamethasone with β-Cyclodextrin and Molecular Docking Study of Its Potential Activity against COVID-19

**DOI:** 10.3390/molecules26247622

**Published:** 2021-12-15

**Authors:** Youghourta Belhocine, Seyfeddine Rahali, Hamza Allal, Ibtissem Meriem Assaba, Monira Galal Ghoniem, Fatima Adam Mohamed Ali

**Affiliations:** 1 Department of Petrochemical and Process Engineering, Faculty of Technology, 20 August 1955 University of Skikda, El Hadaik Road, P.O. Box 26, Skikda 21000, Algeria; im.assaba@univ-skikda.dz; 2 Department of Chemistry, College of Science and Arts, Qassim University, Ar Rass, Saudi Arabia; 3 Department of Technology, Faculty of Technology, 20 August 1955 University of Skikda, El Hadaik Road, P.O. Box 26, Skikda 21000, Algeria; 4 Department of Chemistry, College of Science, Imam Mohammad Ibn Saud Islamic University (IMSIU), Riyadh 11432, Saudi Arabia; mghoniem1@gmail.com (M.G.G.); Famohamedali@imamu.edu.sa (F.A.M.A.)

**Keywords:** β-cyclodextrin, dexamethasone, DFT-D4, molecular docking, non-covalent interactions, COVID-19

## Abstract

The encapsulation mode of dexamethasone (Dex) into the cavity of β-cyclodextrin (β-CD), as well as its potential as an inhibitor of the COVID-19 main protease, were investigated using density functional theory with the recent dispersion corrections D4 and molecular docking calculations. Independent gradient model and natural bond orbital approaches allowed for the characterization of the host–guest interactions in the studied systems. Structural and energetic computation results revealed that hydrogen bonds and van der Waals interactions played significant roles in the stabilization of the formed Dex@β-CD complex. The complexation energy significantly decreased from −179.50 kJ/mol in the gas phase to −74.14 kJ/mol in the aqueous phase. A molecular docking study was performed to investigate the inhibitory activity of dexamethasone against the COVID-19 target protein (PDB ID: 6LU7). The dexamethasone showed potential therapeutic activity as a SARS CoV-2 main protease inhibitor due to its strong binding to the active sites of the protein target, with predicted free energy of binding values of −29.97 and −32.19 kJ/mol as calculated from AutoDock4 and AutoDock Vina, respectively. This study was intended to explore the potential use of the Dex@β-CD complex in drug delivery to enhance dexamethasone dissolution, thus improving its bioavailability and reducing its side effects.

## 1. Introduction

Dexamethasone is a synthetic glucocorticoid—a cheap and well-known drug approved by the FDA in 1958 [1] for which pharmacokinetics studies are well-established—that presents anti-inflammatory and immunosuppressive properties [2,3]. In the medical field, it has a wide variety of uses and has been approved as a therapy of acute exacerbation of inflammatory and respiratory diseases including acute respiratory distress syndrome (ARDS) [4], bacterial meningitis [5], tuberculous meningitis [6], and multiple myeloma [7]. Thanks to its potential to reduce lung inflammation and thus decrease ARDS severity [8], dexamethasone was the first medication to show efficiency in saving lives of coronavirus disease patients [9].

Several randomized clinical trials (RCTs) have been carried out in the United Kingdom [10], Brazil [11], Argentina [12], and other countries using dexamethasone to assess its effectiveness in reducing the mortality risk of patients. A large-scale randomized study comparing 2104 patients receiving the oral or intravenous injection of dexamethasone (6 mg once per day for 10 days with 4321 patients receiving standard care [13,14]) was conducted in the United Kingdom (the RECOVERY trial) for hospitalized patients. The results from the RECOVERY trial indicated that dexamethasone treatment helped to reduce mortality in patients that were mechanically ventilated by one-third and by one-fifth in patients only getting oxygen without invasive mechanical ventilation. Moreover, the efficacy of dexamethasone was only proven for patients requiring respiratory support—not for milder cases where it was not significant [15,16].

In addition to their side effects, corticosteroids and, more specifically dexamethasone, are hydrophobic drugs that represent a real limitation in the achievement of therapeutic concentrations when orally administrated, leading to reduction of the bioavailability of these drugs. Thus, to enhance the aqueous solubility and bioavailability of dexamethasone and to reduce its side effects, the alternative strategy that could be more effective comprises its inclusion in macrocyclic host molecules such as cyclodextrins (CDs) to induce changes in the physicochemical properties of the drugs (guest molecules) [17,18].

CD hosts are a class of natural cyclic oligosaccharides made up of 6–12 glucopyranose units linked by α-(1,4) linkages; the three main common forms called α, β, and γ-cyclodextrins are composed, respectively, of six, seven, and eight D-glucopyranose units [19]. CDs are truncated cone-like structures with a hydrophilic outer surface and hydrophobic inside cavity, allowing them to be soluble in water and able to occlude hydrophobic guest molecules [19,20]. Due to their advantageous properties of non-toxicity, facile modification, good water-solubility, and high biological availability, CDs have gained tremendous attention and found versatile applications, mainly in the food, pharmaceutical, and cosmetic industries [21,22]. Particularly, CDs and β-CD have been extensively studied as useful functional pharmaceutical excipients, especially as drug-delivery systems [23]. Due to the hydrophobicity of CD cavities, they can effectively interact with many poorly soluble drugs to form inclusion complexes, resulting in the enhancement of the aqueous solubility, bioavailability, and physicochemical stability of drugs [24,25].

In this context, in the context of dental pulp therapy, Daghrery et al. [26] experimentally developed a drug-delivery system based on the formation of an inclusion complex between dexamethasone and β-CD to assess the mineralization capacity of stem cells from human-extracted deciduous teeth (SHEDs). The authors evidenced a significant enhancement of DEX solubility, a higher release, a decrease in SHED toxicity, and a significant increase in mineralization. Vianna et al. [17] experimentally studied the inclusion of dexamethasone acetate (DMA) in β-, γ-, and hydroxypropyl-β-CD (HP-β-CD), and they showed that the formation of CD inclusion complexes with DMA may be an interesting approach for drug-delivery applications. Additionally, the authors performed basic molecular modeling using force field calculations and proposed some molecular models of the DMA@βCD inclusion complex; however, the geometry and driving forces involved in the inclusion process were not well-elucidated.

Computational chemistry simulations are an efficient tool that can provide valuable insight into the inclusion process and the nature of non-covalent interactions occurring in host–guest complexes [27,28,29,30,31,32]. In particular, density functional theory (DFT) is well-suited for investigating these systems and predicting their energetic and structural properties [33,34,35].

The current theoretical study, exclusively based on the DFT approach, aimed to provide a more profound understanding of the inclusion complex formation between dexamethasone (Dex) and β-CD. The most recent development of DFT-based methods consisting of the use of the new D4 empirical dispersion correction was applied for the evaluation of the energetic and structural properties of the Dex@β-CD complex. Furthermore, docking studies were carried out to explore and analyze the binding affinity and interactions between dexamethasone and the SARS-CoV-2 target protein (6LU7).

## 2. Results and Discussion

### 2.1. DFT-D4 Calculations of Complexation Energies

The values of the computed complexation energy in gas and aqueous phases as a function of the position coordinate of each configuration on the Z-axis for the A and B modes are reported in Table 1. All optimized configurations were found to be associated with negative complexation energy, indicating that a thermodynamically favorable process occurred. Among the studied configurations, the lowest energy structure corresponding to the most stable configuration was found to be located at Z = 6 Å for A mode, and it had the largest complexation energy (−179.50 kJ/mol).

The structural re-optimization of the most stable complex in aqueous solution using an SMD solvation model decreased the complexation energy to −74.14 kJ/mol, suggesting that the complexation process was more stable in the gas phase.

**Figure 1:**
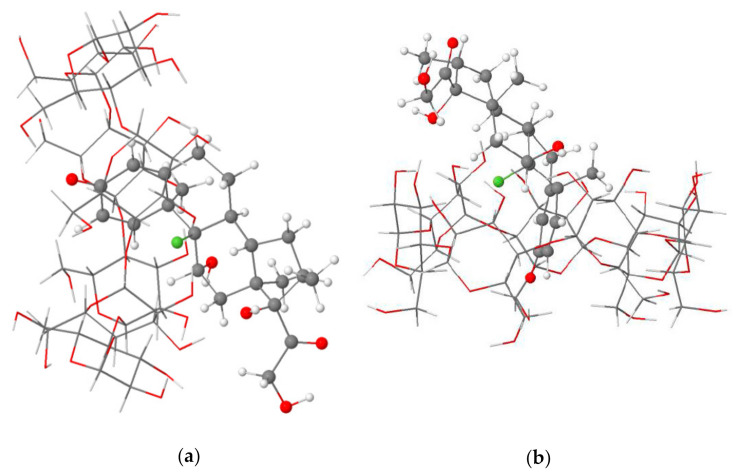
Side (**a**) and top (**b**) views of the partial inclusion of dexamethasone in the β-CD cavity as calculated at the BLYP-D4/def2-TZVP level of theory in the gas phase.

The structural analysis of the most stable Dex@β-CD complex in the gas phase and an aqueous solution indicated the partial inclusion of the dexamethasone in the β-CD cavity from the wider rim (mode A) through its cyclohexadienone moiety, as shown in Figure 1. A graphical animation of the inclusion process is provided as Supplementary Materials (animated GIF file).

### 2.2. Analysis of the Non-Covalent Intermolecular Interactions

The study of the role and the nature of non-covalent intermolecular interactions provided an effective tool for the identification of the mechanisms involved in the stabilization of the Dex@β-CD complex. The intermolecular interactions are denoted in the IGM plots by blue and green colored areas that, respectively, correspond to hydrogen-bond interactions and weak dispersive forces.

**Figure 2:**
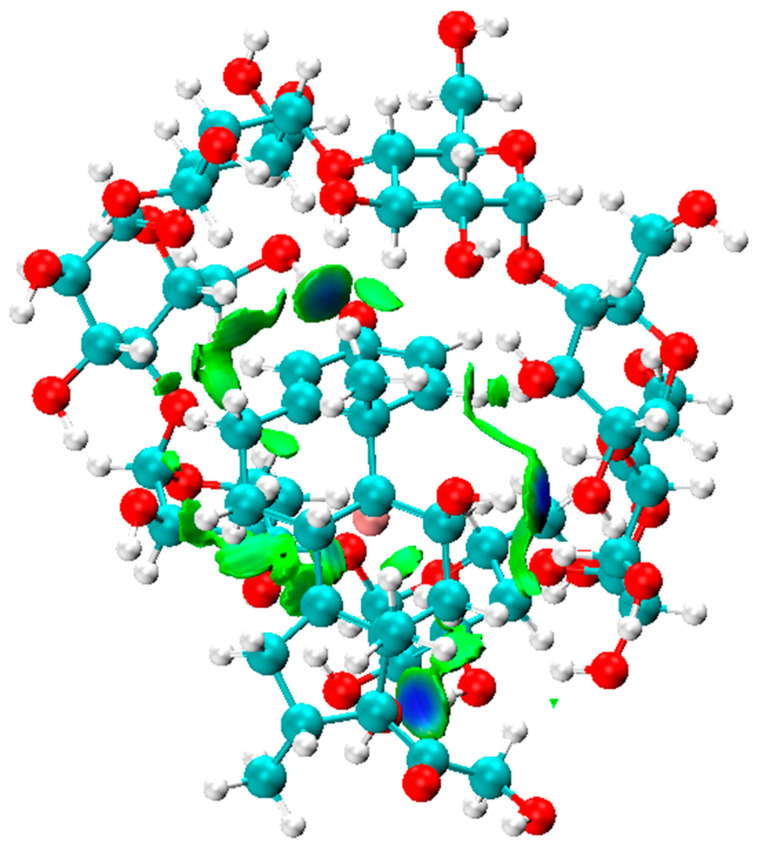
The IGM isosurface (isovalue = 0.006 a.u.) of the Dex@β-CD complex.

As shown in Figure 2, the IGM isosurface plot (0.006 a.u.) of the Dex@β-CD complex was mainly dominated by three blue discs associated with intermolecular hydrogen bonds and green areas that characterize weak attractive interactions that indicate the role of weak Van der Waals interactions and hydrogen bonding in the formation and stabilization of the Dex@β-CD complex.

### 2.3. Contribution of Intermolecular Hydrogen Bonds

To estimate the contribution of hydrogen bonds to the stabilization of the Dex@β-CD complex, a natural bond orbital calculation (NBO) [36] was conducted with Gaussian 09 code [37] on the optimized water-phase structure using the M06-2X functional [38] and def2-TZVPP basis set [39,40]. The significant occurring intermolecular hydrogen bonds (>10 kJ/mol) computed using donor–acceptor interaction energies (E^(2)^) through NBO analysis are reported in Table 2.

As can be seen in Table 2, the intermolecular hydrogen bonds mainly occurred between oxygen lone-pair electron-donating orbitals (LP) and σ* (BD*) O–H antibonding orbitals. The strongest H-bonds with higher stabilization energies (63.39, 19.87, 37.66, and 19.41 kJ/mol) were found to correspond to the shortest H-bonds (1.80, 1.79, 1.79, and 1.90 Å), and these interactions were found to be associated with the blue discs of the IGM isosurface that indicate the presence of the intermolecular hydrogen bonds, therefore confirming the role of hydrogen-bonding interaction [33,41] in the formation and stabilization of the Dex@β-CD complex. It is worth noting the presence of a weak hydrogen bond (10.67 kJ/mol) corresponding to the interaction between the lone-pair electron-donating orbitals LP(3) of fluorine (F148) and the anti-bonding orbital of the O45–H59 bond of β-CD.

**Figure 3:**
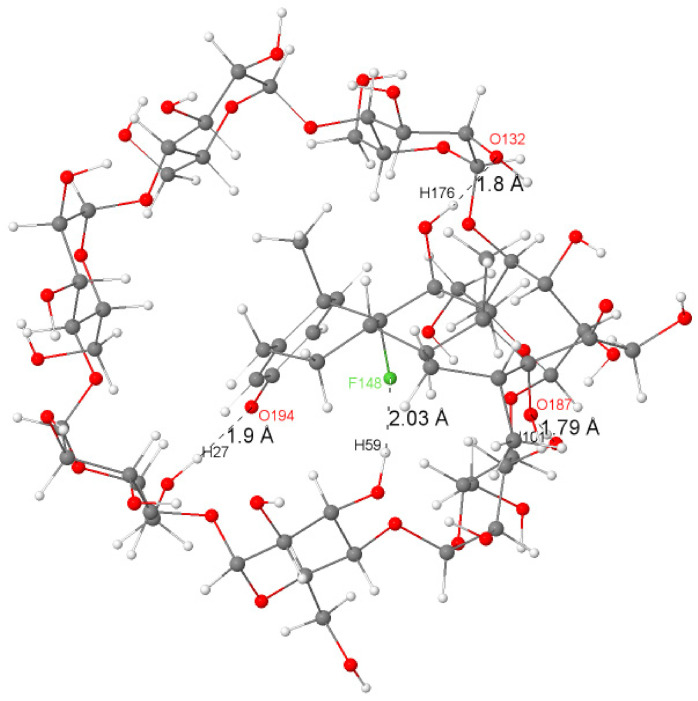
The significant H-bonds (in angstrom) between dexamethasone and β-CD in the Dex@β-CD complex.

Figure 3 illustrates a graphical representation of the most important intermolecular hydrogen bonding in the structure of the Dex@β-CD complex.

### 2.4. AutoDock Docking Result Analysis

**Figure 4:**
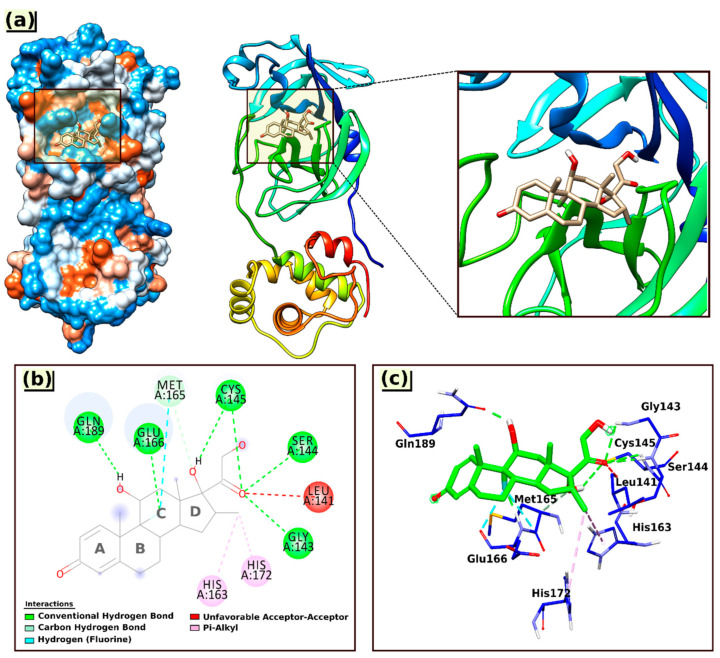
3D visual representations of the interactions of Dex with the 6LU7 protein (**a**) best binding mode in the protein pocket; (**b**,**c**) amino acid residues involved in the interaction.

The analysis map results and calculated parameters of the most stable docked pose for the interactions of Dex with the 6LU7 protein are shown in Figure 4 and Table 3, respectively, and the interacting amino acid residues are summarized in Table 4.

Based on the docking results (Table 3), it can be concluded that Dex bound strongly to the active sites of the protein target, with predicted free energy of binding (BE) values of −29.97 and −32.19 kJ/mol, respectively, obtained by employing AutoDock4 and AutoDock Vina; the estimated inhibition constant (KiC) value was 5.59 uM.

As shown in Figure 4, the Dex formed one carbon–hydrogen bond with Met165(A) via an aromatic ring (ring C); six conventional hydrogen bonds with the nearest amino acid residues Gln189(A), Glu166(A), Cys145(A), Ser144(A), and Gly143(A), among which twice with Cys145(A) through the hydroxyl group at ring D and the carbonyl group of the ketone moiety. All the hydrogen bond distances were observed within the range from 1.77 to 3.36 Å.

In addition to hydrogen bonding, the interaction of Dex at the active site of 6LU7 also involved an unfavorable acceptor–acceptor interaction with Leu141(A) (2.55 Å) and two hydrophobic interactions (Pi–alkyl) with amino acid residues His172(A) and His163(A), with bond distances of 5.38 and 4.40 Å, respectively (Table 4).

Several studies have been conducted to investigate the inhibition ability of bioactive compounds and natural products against the COVID-19 main protease (6LU7) using molecular docking simulations [42,43,44,45,46]. Chhetri et al. [42] examined a series of six novel imidazole anchored azo-imidazole derivatives to ascertain their inhibitory activity on the main protease (6LU7) and concluded from the results of docking calculations that all studied compounds exhibited a significant inhibitory effects, with the binding energies ranging from −33.89 to −28.03 kJ/mol. Rangsinth et al. [46] examined 36 bioactive compounds for their potential as SARS-CoV-2 main protease inhibitors. According to the calculated binding energies that varied between −44.14 and −17.82 kJ/mol, they established that 25 of the 36 candidate compounds could inhibit the main viral protease.

## 3. Computational Procedure

### 3.1. DFT Calculations

**Figure 5:**
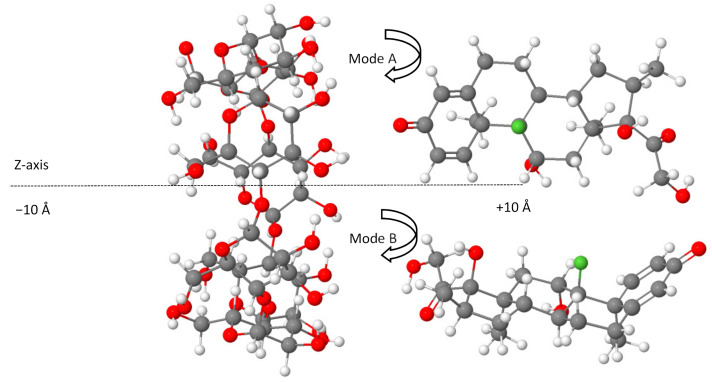
The generated initial set of configurations for a dexamethasone docking prediction from −10 Å to +10 Å for A and B modes. Color code: carbon, grey; fluorine, green; oxygen, red; hydrogen, white.

Density functional theory computations were carried out using the ORCA program (version 4.2.1) [47,48]. The full geometry optimization of the structures was conducted in the gas phase by employing the BLYP-D4 functional [49,50,51] coupled with the def2-TZVP basis set [39]. Due to the size of the studied systems (204 atoms and 812 electrons for each configuration of non-covalent complexes), the resolution of the identity method was applied to speed up the calculations [40,52]. The use of the most recent dispersion-corrected DFT approximation based on the new charge-dependent D4 dispersion model is an adequate approach for describing the interactions of non-covalent systems [53,54]. The starting configurations used for molecular docking simulations between β-CD and dexamethasone were generated according to the method of Liu and Guo [55], where the center of dexamethasone and β-CD was defined as the center of the coordination system (0 Å). The axis of the dexamethasone was directed along the Z-axis of β-CD, on which the dexamethasone was translated from −10 to +10 Å with a step of 2 Å (Figure 5), resulting in two possible modes of complex inclusion in which the dexamethasone approached the wider rim of the β-CD cavity by its cyclohexadienone group (mode A) or through the terminal oxo and hydroxy groups from the opposite side (mode B), as represented in Figure 5 using the Jmol viewer applet [56].

The complexation energies for all created configurations were computed using Equation (1):

(1) $$\Delta E_{\mathrm{complexation}}=E_{\mathrm{Dex}@\beta -\mathrm{CD}}-(E_{\mathrm{Dex}}+E_{\beta -\mathrm{CD}})$$

where ∆E_complexation_ represents the complexation energy, and E _Dex@β-CD_, E _Dex_, and E _β-CD_ are, respectively, the optimized energies of the complex, the free dexamethasone, and the free β-CD.

The obtained most stable configuration corresponding to the lowest energy structure was re-optimized in a water solvent through the SMD solvation model [57] at the BLYP-D4/def2-TZVP level of theory.

The re-optimized aqueous phase structure was then used to further analyze the intermolecular interactions, such as non-covalent interactions (NCIs), based on the independent gradient model (IGM) analysis [58] with the help of the wave function analysis code Multiwfn [59] and the VMD visualization program [60].

### 3.2. Molecular Docking Study Using AutoDock

The 3D crystal of the main protease of SARS-CoV-2 (PDB ID: 6LU7) in complex with an N3 inhibitor was downloaded from the Protein Data Bank (PDB) (https://www.rcsb.org accessed on 13 October 2021) [61], and we used the geometry of the previously optimized structure for the dexamethasone ligand in this study. The docking studies were performed using AutoDock4 and AutoDock Vina implemented in AutoDockTools (ADT 1.5.6) software [62]. All water molecules, ligands, and ions were cleaned (removed) from the PDB file of 6LU7 using UCSF Chimera software (ver. 1.10.2) [63]. The non-polar hydrogens were merged, and then the Kollman partial charges [62] were assigned using AutoDockTools. The docking box with an 80 × 80 × 80 Å grid was defined and employed with a grid spacing of 0.375 Å. The most representative docked poses were visualized using CHIMERA (UCSF) [63] and BIOVIA Discovery studio visualizer (version 1.10.2) [64], in which the root mean square deviation (RMSD) was less than 2 Å.

## 4. Conclusions

The host-guest inclusion process of dexamethasone into β-CD was studied using the DFT-D4 approach. Molecular docking simulations were also conducted to assess the potential inhibitory activity of dexamethasone against SARS-CoV-2 by targeting its main protease. The analysis of structural, energetic, and electronic properties using IGM and NBO allowed us to characterize the nature of the host–guest interactions in the Dex@β-CD complex. The results showed that dexamethasone partially penetrates the cavity of β-CD from the wider rim through its cyclohexadienone moiety. The complexation energy in the gas phase was found to be −179.50 kJ/mol and decreased to −74.14 kJ/mol in the aqueous phase. Hydrogen bonds and Van der Waals interactions were found to be mainly responsible for the formation of the Dex@β-CD complex. Molecular docking simulations revealed that dexamethasone binds strongly to the active sites of the protein target, and AutoDock and AutoDock Vina predicted free binding energies of −29.97 and −32.19 kJ/mol, respectively. This study has shown that the inclusion complexation of dexamethasone with β-CD could be an adequate pharmaceutical strategy to overcome its lower solubility and improve its bioavailability, thus enhancing its therapeutic potential against SARS-CoV-2 infection while reducing its side effects.

## Figures and Tables

**Table 1:** Complexation energies (in kJ/mol) of β-CD with dexamethasone calculated in the gas phase at BLYP-D4/def2-TZVP level.

Inclusion Configurations	Mode A	Mode B
−10	−101.30	−100.70
−8	−104.96	−91.19
−6	−94.04	−134.17
−4	−115.24	−115.77
−2	−162.29	−142.11
0	−161.26	**−174.67**
2	−137.76	−147.21
4	−153.71	−107.88
6	**−179.50**	−164.56
8	−175.09	−137.95
10	−175.42	−116.61

**Table 2:** NBO analysis of hydrogen-bonding interactions and stabilization energies E^(2)^ (kJ/mol) for the Dex@β-CD complex.

Complex	Donor	Acceptor	H-bond (Å)	E^(2)^ (kJ/mol)
Dex@β-CD	β-CD (Donor)	Dex (Acceptor)		
	LP(2) O132	BD*(1) O164–H176	1.80	63.39
	Dex (Donor)	β-CD (Acceptor)		
	LP(3) F148	BD*(1) O45–H59	2.03	10.67
	LP(1) O187	BD*(1) O87–H101	1.79	19.87
	LP(2) O187	BD*(1) O87–H101	1.79	37.66
	LP(1) O194	BD*(1) O20–H27	1.90	19.41
	LP(2) O194	BD*(1) O20–H27	1.90	12.30

BD* denotes σ* antibonding orbital, and LP denotes lone valence pair.

**Table 3:** Calculated parameters ^(a–e)^ of docked Dex with 6LU7 protein.

BE ^a^		KiC ^b^	TIE ^c^	FIE ^d^	EE ^e^
AutoDock4	AutoDock Vina				
−29.97	−32.19	5.59	−36.20	−3.55	−0.59

BE ^a^: free energy of binding (kJ/mol). KiC ^b^: estimated inhibition constant, Ki. (uM: micromolar). ^c^ TIE: total intermolecular energy (kJ/mol). FIE ^d^: final total internal energy (kJ/mol). EE ^e^: electrostatic energy (kJ/mol).

**Table 4:** Amino acid contributions in the interactions of Dex with the 6LU7 protein.

	Amino Acids Involved in the Interactions (Interaction Site)	Distances (Å)
6LU7@Dex	Gln189(A), Glu166(A), Cys145(A), Ser144(A), Gly143(A), Met165(A), His172(A), His163(A), and Leu141(A).	Lig−Glu166(A) (1.77, 3.08) Lig−Gln189(A) (1.80) Lig−Cys145(A) (2.91, 2.91) Lig−Ser144(A) (2.33) Lig−Gly143(A) (2.70) Lig−Met165(A) (3.36) Lig−His172(A) (5.38) Lig−His163(A) (4.40) Lig−Leu141(A) (2.55)

## Data Availability

The data presented in this study are available on request from the corresponding author.

## Supplementary Materials

The following are available online, A graphical animation (animated GIF file) of the inclusion complexation of dexamethasone in β-CD calculated at the BLYP-D4/def2-TZVP level in the gas phase is provided.
