# Supplementary figures and images for: A Dispersion Corrected DFT Investigation of the Inclusion Complexation of Dexamethasone with β-Cyclodextrin and Molecular Docking Study of Its Potential Activity against COVID-19
Source: Molecules. 2021 Dec 15;26(24):7622. doi: 10.3390/molecules26247622 (PMC8708408; doi:10.3390/molecules26247622)

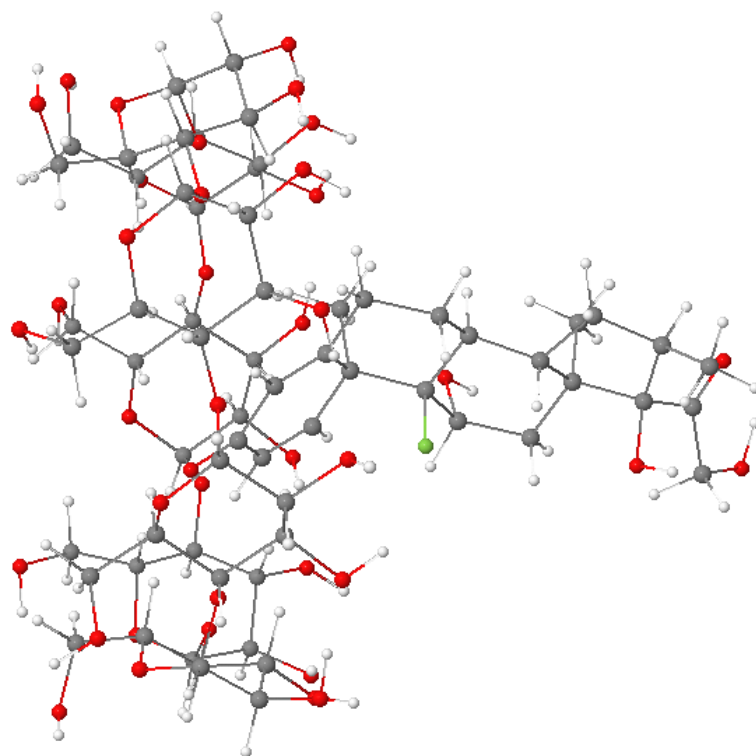

Jmol

Supplement: Supplementary file 1 [file molecules-26-07622-s001.zip › molecules-1485390-supplementary.pdf]
